# Ophthalmology

**Published:** 1878-08

**Authors:** 


### Ophthalmology.

Drainage for Detached Retina.—Cohn. (*Deut. Zeit. f. Pr. Med.*)

The author gives a summary of our knowledge and treatment of this disease. Arlt and others have tried to cure it by piercing the sclerotic. Graefe did this and divided the retina. But inflammation is liable to follow either of these measures. Pressure and confinement in a dark room occasionally yield good results. In 1877 Wecker suggested the introduction through the sclerotic, and under the retina of a gold thread as a means of drainage. In four cases Cohn has tried it, and with excellent results. As soon as the retina becomes attached again, perception is restored, and this has happened even after a lapse of three years; the perception of space only, however, and not that of color. In no case was any inflammation aroused, or any harm done; nor does the method interfere with other treatment.

